# Class 1 integron and Imipenem Resistance in Clinical Isolates of *Pseudomonas aeruginosa*: Prevalence and Antibiotic Susceptibility

**Published:** 2010-09

**Authors:** S Yousefi, MR Nahaei, S Farajnia, M Ghojazadeh, MT Akhi, Y Sharifi, M Milani, R Ghotaslou

**Affiliations:** 1 Drug Applied Research Center, Tabriz University of Medical Sciences, Tabriz, Iran; 2 Microbiology Department, Faculty of Medicine, Tabriz University of Medical Sciences, Tabriz, Iran; 3 Biotechnology Research Center, Tabriz University of Medical Sciences, Tabriz, Iran; 4 Physiology Department, Faculty of Medicine, Tabriz University of Medical Sciences, Tabriz, Iran; 5 Clinical microbiology laboratory of Imam Hospital, Orumieh University of Medical Sciences

**Keywords:** Carbapenem, drug resistance, *Pseudomonas aeruginosa*, class 1 integron

## Abstract

**Background and Objectives:**

*Pseudomonas aeruginosa* is one of the most important causative agents of nosocomial infections especially in ICU and burn units. *P. aeruginosa* infections are normally difficult to eradicate due to acquired resistance to many antibiotics. Recent appearance of carbapenem resistant *P. aeruginosa* isolates is considered a major healthcare problem. The present study was conducted to detect class 1 integron and antibiotic susceptibility profiles of imipenem-sensitive and resistant clinical isolates of *P. aeruginosa*.

**Materials and Methods:**

Antibiotic susceptibility profiles and minimum inhibitory concentration against imipenem was studied in 160 clinical isolates of *P. aeruginosa* by disk agar diffusion method and Etest, respectively. Detection of class 1 integron was performed by the PCR method. Demographic and microbiological data were compared between imipenem susceptible and non-susceptible isolates by the SPSS software.

**Results:**

PCR results showed that 90 (56.3%) of *P. aeruginosa* isolates carried class 1 integron. Antibiotic susceptibility results revealed that 93 (58.1%) were susceptible and 67 (41.9%) were non-susceptible to imipenem. Comparison of antibiotic susceptibility patterns showed high level of drug resistance among imipenem non-susceptible isolates. We found that MDR phenotype, presence of class 1 integron and hospitalization in ICU and burn units were significantly associated with imipenem non-susceptible isolates.

**Conclusion:**

The high frequency of imipenem resistance was seen among our *P. aeruginosa* isolates. Since carbapenems are considered as the last drugs used for treatment of *P. aeruginosa* infections, it is crucial to screen imipenem non-susceptible isolates in infection control and optimal therapy.

## INTRODUCTION

*Pseudomonas aeruginosa* is a ubiquitous Gram-negative bacterium causing various types of infections and frequently isolated from clinical specimens (1). The range of diseases caused by this bacterium varies from superficial skin infections to serious systemic infections such as fulminant sepsis (2). *P. aeruginosa* is the second most common causative agent of hospital-acquired pneumonia, healthcare-associated pneumonia and ventilator-associated pneumonia. *P. aeruginosa* is also an important pathogen in immunocompromised patients, such as patients suffering from AIDS, cancer, burn wounds and cystic fibrosis (CF) (3). Infections caused by *P. aeruginosa* are often difficult to eradicate because it requires minimal nutrition and can tolerate a wide range of temperatures. Also, it is resistant to many antibiotics, disinfections and has the ability to acquire resistance (4).

Carbapenem antibiotics remain as the last the rapeutic option for treatment of serious infections caused by *P. aeruginosa*. Recently, emerging antimicrobial resistance to most classes of antibiotics, including carbapenems, resulted in developing multidrug resistant *P. aeruginosa* isolates (isolates resistant to at least 3 different classes of antibiotics) (5). Emergence of multiple drug resistance *P. aeruginosa* appeared as a great problem in clinical settings due to limited therapeutic options (6, 7). Various mechanisms are involved in antimicrobial resistance in *P. aeruginosa* including intrinsic resistance and gene acquisition by horizontal transfer mediated by mobile genetic elements., Plasmids, transposons and integrons are vehicles and structures for mobilization, acquisition and spreading of resistance genes (8). Integrons are common gene capture and expression systems that incorporate ORFs (open reading frame) and convert them into functional genes. The essential components of an integron include the integrase gene (*int* I), the attachment site (*att* I) and the promoter, which promotes the expression of any suitably integrated gene (s) (9).

Multiple classes of integrons have been identified according to their distinct integrase genes in Gram-negative bacteria. Class 1 integrons are the most prevalent in clinical isolates and carry single or multiple gene cassettes. Genes carried by integrons encode for various antibiotic resistance mechanisms, including resistance to aminoglycosides, sulphonamids, β-lactams, macrolides, chloramphenicol, antiseptics and disinfectants. In case of β-lactamases, integron-born gene cassettes have been found mainly in *P. aeruginosa*, *Acinetobacter baumannii* and various species of Enterobacteriaceae (10), but its prevalence is variable in different parts of the world (8, 11, 13). The present study was designed to investigate the prevalence of class 1 integron and antimicrobial susceptibility profiles of imipenem susceptible and imipenem non-susceptible isolates of *P. aeruginosa* at a University Hospital in Orumieh, Iran.

## MATERIALS AND METHODS

**Bacterial isolates.** A total of 160 non-duplicate clinical isolates of *P. aeruginosa* isolates were collected from Orumieh Imam Hospital between August 2007 and August 2008. A questionnaire was used for recording patients’ demographic and clinical data. Bacterial isolates were recovered from different clinical samples such as; bronchial fluid, blood culture, catheter, cerebrospinal fluid, ear, pleural fluid, sputum, urine and wound. All isolates were identified as *P. aeruginosa* by using standard microbiological tests such as; Gram stain, oxidase test, growth at 42°C, growth on Cetrimide agar medium (Liofilchem, Italy), O/F (Oxidation-Fermentation) test and pigment production (14).

**Antimicrobial Susceptibility testing.** Antimicrobial susceptibility testing was performed by the disk agar diffusion method according to Clinical and Laboratory Standards Institute (CLSI) recommendations (15).

The following antimicrobial disks (Mast Co. UK) were used for antimicrobial susceptibility testing; imipenem (10 µg), meropenem (30 µg), aztreonam (10 µg), cefepime (30 µg), ceftazidime (30 µg), ceftriaxone (30 µg), cephalexin (10 µg), amikacin (30 µg), gentamicin (10 µg), ciprofloxacin (30 µg), norfloxacin (10 µg), piperacillin/tazobactam (100 µg). *P. aeruginosa* ATCC 27853 was used as quality control in each run of antimicrobial susceptibility testing.

**Determination of minimum inhibitory concentration.** Minimum inhibitory concentrations (MICs) of imipenem susceptible and imipenem non-susceptible isolates of *P. aeruginosa* were determined by using Etest strips (BioMerieux AB Biodisk Solna-Sweden). The procedure was as follows; fresh colonies from overnight culture of bacterial isolates were used for preparation of 0.5 McFarland turbidity standard. Then bacterial suspension was inoculated onto the Muller-Hinton agar (Merck, Germany) plates by using a cotton swab and spread all over the plate. Imipenem Etest strip was laid on the surface of Muller-Hinton agar plate in a position that the whole length of strip be in complete contact with the agar surface. After incubation at 37°C for 16–18 hours, the MIC values were read where the inhibition ellipses intersect the Etest strip. *P. aeruginosa* ATCC 27853 was used as a control for antimicrobial susceptibility testing.

**DNA extraction.** DNA extraction was performed by simple boiling method. In practice, 2–3 colonies of overnight culture of *P. aeruginosa* isolates were suspended in 400 µl of 1× TE buffer (10 mM Tris, 1m M EDTA) by vortexing. The suspension was heated in a boiling bath at 95°C for 10 min. Then cellular debris was removed by centrifugation at ×12000 RPM for 10 min. One µl of supernatant was used as template DNA in PCR reactions.

**Class 1 integron PCR.** The integrase gene (*int* I) was amplified in PCR reaction for detection of class 1 integron using primers: *Int*1-F: 5-GGTGTGGCGGGCTTCGTG-3 and *Int*1-R: 5-GCATCCTCGGTTTTCTGG-3 (16). PCR master mix component was as follows; 10× PCR buffer in final concentration of 1×, MgCl_2_ (50 mM) in a final concentration of 1.5 mM, dNTP Mix, 10 mM in a final concentration of 0.2 mM, forward and reverse primers in a final concentration of 0.4 µM. PCR amplification was performed in a total volume of 25 µl (24 µl of PCR master mix plus 1 µl of template DNA). *P. aeruginosa* PA66 (VIM positive) and *Serratia marcescens* CI 10-4–9 (IMP positive) isolates were used as positive controls for class 1 integron.

PCR amplification condition was as follows: initial denaturation at 95°C for 4 min followed by 35 cycles of 60 seconds at 94°C (denaturation), 60 seconds at 62°C (annealing) and 45 seconds at 72°C (extension) with a final extension at 72°C for 7 minutes. PCR products were analyzed by electrophoresis in 1.2% agarose gel in a TAE buffer at 90 volts alongside with 1 Kbp DNA ladder. Then gels were stained in ethidium bromide solution for 15 minutes and finally visualized in gel documentation system.

**Statistical analysis.** For statistical analysis, the isolates were divided into two groups; imipenem susceptible isolates and imipenem non-susceptible isolates. Descriptive statistics including frequencies, cross-tabulation of microbiological, clinical and demographic data were analyzed using SPSS statistical software (version 16.0). The χ^2^ test, or the Fishers’ exact test, when appropriate, was used in a univariate analysis to assess the differences between two groups of isolates, *p* values less than 0.05 were considered statistically significant.

## RESULTS

During the study period, a total of 160 isolates of *P. aeruginosa* were collected from different wards of Orumieh Imam Hospital which is a tertiary care center of Orumieh Medical Sciences University. One hundred and forty six (91.2%) of isolates were obtained from hospitalized patients and 14 (8.8%) belonged to out-patients. Frequency of isolates according to hospital wards were as follows; burn unit 52 (32.5%), ICU wards 32 (20%), surgery wards 18 (11.2%), nephrology ward 14 (8.8%), internal medicine wards 13 (8.1%), urology ward 7 (4.4%), kidney transplant unit 5 (3.1%), oncology ward 5 (3.1%) and 14 (8.8%) were isolated from out-patients.

**Fig. 1:**
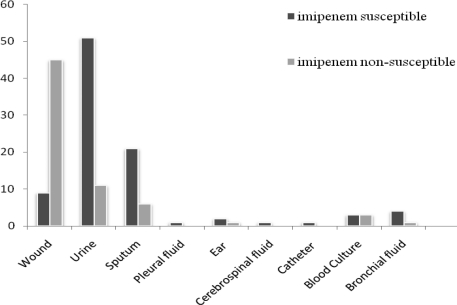
Specimen sources of imipenem susceptible and non-susceptible *P. aeruginosa* isolates.

Specimen sources with regard to the two categories of imipenem susceptible and non-susceptible isolates have been demonstrated in Fig. 1. Mean age of patients was 42±25.1 (rang: 1–111). Ninety-seven (60.6%) patients were males and 63 (39.4%) patients were females (male to female ratio 1.54:1).

Antibiotic susceptibility results showed that 93 (58.1%) of *P. aeruginosa* isolates were sensitive to imipenem, 61 (38.1%) were resistant and 6 (3.8%) of isolates showed intermediate resistance. Antibiotic susceptibility testing showed that overall rate of drug resistance among imipenem non-susceptible isolates was higher than imipenem susceptible isolates. These differences were statistically significant (Table 1). Determination of MIC against imipenem by Etest method revealed that Mean MIC among the imipenem non-susceptible isolates was 31.43 mg/L while this value for imipenem susceptible isolates was 2.86 mg/L.

**Table 1:** Antibiotic susceptibility in imipenem susceptible and non-susceptible *P. aeruginosa* isolates.

Antibiotic	Imipenem susceptible (n=93)						Imipenem non-susceptible (n=67)					
	
	Sensitive		Intermediate		Resistant		Sensitive		Intermediate		Resistant	
	No.	(%)	No.		No.	(%)	No.	(%)	No.	(%)	No.	(%)
**Amikacin**	73	(78.5)	1	(1.1)	19	(20.4)	6	(9)	-	-	61	(91)
**Aztreonam**	40	(43)	24	(25.8)	29	(31.2)	3	(4.5)	3	(4.5)	61	(91)
**Ceftazidime**	68	(73.1)	2	(2.1)	23	(24.7)	8	(11.9)	3	(4.5)	56	(83.6)
**Ciprofloxacin**	58	(62.4)	2	(2.1)	33	(35.5)	5	(7.5)	2	(3)	60	(89.5)
**Cephalexin**	35	(37.6)	2	(2.1)	56	(60.2)	5	(7.5)	1	(1.5)	61	(91)
**Cefepime**	65	(69.9)	2	(2.1)	26	(28)	4	(6)	2	(3)	61	(91)
**Ceftriaxon**	14	(15)	6	(6.5)	73	(78.5)	3	(4.5)	3	(4.5)	61	(91)
**Cefotaxime**	35	(37.6)	1	(1.1)	57	(61.3)	3	(4.5)	9	(13.4)	55	(82.1)
**Gentamicin**	58	(62.4)	2	(2.1)	33	(35.5)	4	(6)	2	(3)	61	(91)
**Meropenem**	87	(93.4)	5	(5.4)	1	(1.2)	4	(6)	1	(1.5)	62	(92.5)
**Norfloxacin**	48	(51.6)	4	(4.3)	41	(44.1)	7	(10.4)	4	(6)	56	(83.6)
**Piperacillin/tazobactam**	76	(81.7)	7	(7.5)	10	(10.8)	4	(6)	2	(3)	61	(91)

Analysis of patients’ demographics and microbiological data between imipenem susceptible and non-susceptible isolates by SPSS software showed that majority of imipenem non-susceptible isolates belonged to hospitalized patients harboring class 1 integron and were MDR *P. aeruginosa*. In addition, we found that hospitalization in burn units and ICU wards had significant association with imipenem non-susceptible isolates (*p<*0.05).

**Fig. 2:**
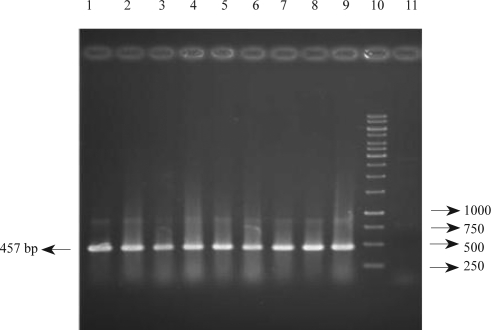
PCR amplification of Class 1 integron gene in clinical *P. aeruginosa* isolated from Imam Hospital of Orumieh, Iran. Lanes; 1: positive isolate, 2: positive isolate, 3–6: positive isolate, 7: positive isolate, 8, 9: positive control isolates (VIM and IMP type positive isolates), 10: 1 Kbp DNA ladder, 11: negative control.

Investigation of class 1 integron by PCR method indicates that 90 (56.3%) of *P. aeruginosa* isolates carried this gene (Fig. 2). Frequency of class 1 integron were 29 (31.2%) and 61 (91.0%) in imipenem susceptible and imipenem non-susceptible isolates, respectively. Other factors associated with imipenem susceptibility among *P. aeruginosa* isolates have been presented in Table 2.

**Table 2:** Demographic, clinical and microbiological data of imipenem susceptible and non-susceptible *P. aeruginosa* isolates.

Demographic, clinical and microbiological variables		Imipenem susceptible (n=93)	Imipenem non-susceptible (n=67)	*p*
**Demographic:**				
Mean age (± SD) 1		50.74±23.88	29.85±21.54	< 0.001
Gender	Female	31 (33.3%)	32 (47.8%)	0.073
	Male	62 (66.7%)	35 (52.2%)		
Patients	In-patients	80 (86%)	66 (98.5%)	0.008
	Out-patients	13 (14%)	1 (1.5%)		
**Clinical:**				
Surgery		48 (51.6%)	41 (61.2%)	0.261
Hospitalized in burn ward		2 (2.2%)	50 (74.6%)	< 0.001
Hospitalized in ICU ward		46 (49.5%)	11 (16.4%)	< 0.001
Chronic lung disease		16 (17.2%)	7 (10.4%)	0.261
Mechanical ventilation		15 (16.1%)	9 (13.4%)	0.823
Death during hospitalization		17 (18.3%)	16 (23.9%)	0.431
**Microbiological:**				
MDR P. aeruginosa		28 (30.1%)	62 (92.5%)	< 0.001
Class 1 integron gene		29 (31.2%)	61 (91%)	< 0.001

## DISCUSSION

*P. aeruginosa* is one of the most common causes of life-threatening and difficult-to-treat nosocomial infections (17). Resistance to antimicrobial agents appeared as a great problem in clinical settings. Recently emerging carbapenem resistance in *P. aeruginosa* isolates have limited therapeutic options for treatment of MDR *P. aeruginosa* which are considered as the last line of drugs for treatment of infections caused by these organisms (18, 19). Different resistance associated encoding genes such as extended spectrum β-lactamases (ESBLs), which hydrolyse third and fourth generation cephalosporins, and metallo-β-lactamases (MBLs), which hydrolyse carbapenems, are located in class 1 integron structures (8).

Since knowledge about, antibiotic susceptibility can help choose the appropriate treatment agents and also to control nosocomial infections, in the present study we investigated the antibiotic susceptibility pattern, presence of class 1 integron and imipenem resistance in clinical isolates of *P. aeruginosa* at Orumieh University Hospital.

The results of the present study showed a high level of antimicrobial resistance among *P. aeruginosa* isolates. Sixty-seven (41.9%) of *P. aeruginosa* isolates were non-susceptible to imipenem. Other studies in Iran reported that the prevalence of imipenem resistance *P. aeruginosa* varied from 2.9% to 61.83% in Tehran, the capital of Iran (20, 21). Saderi *et al.*, reported that 38.28% of *P. aeruginosa* isolates from Tehran were resistant to imipenem (22), Khosravi *et al*. reported that 41% of *P. aeruginosa* isolates from burn patients in Ahwaz (located in southwest of Iran) showed resistant to imipenem (23).

Comparison of antibiotic susceptibility pattern between imipenem susceptible and imipenem non-susceptible isolates revealed that the resistance rate among imipenem non-susceptible isolates were higher than imipenem susceptible isolates. This difference was statistically significant (p<0.05) in case of all tested antibiotics except ceftriaxone. Imipenem, meropenem and piperacillin/tazobactam were the most effective antibiotics respectively. This was in accordance with findings of Japoni and coworkers in south of Iran (24).

Previous studies have shown that antibiotic resistance rate in clinical settings is higher which is probably because of wide range of antibiotic use in hospitalized patients. In our study, 91.25% of isolates belonged to the hospitalized patients from which 52.5% of isolates were collected from ICU and burn units (19, 25). Furthermore, in our study, all hospitalized patients in ICU and burn units had a history of imipenem administration during their hospital stay. This also was reported by Onguru *et al*. It seems prior exposure to antibiotics remains as one of the most important factors for drug resistance (26).

Screening of *P. aeruginosa* isolates for class 1 integron revealed that there is a significant difference among imipenem susceptible and imipenem non-susceptible isolates. These data are in accordance with Fonseca *et al*. findings that reported 56.6% of imipenem non-susceptible and 32.5% of imipenem susceptible isolates were positive for class 1 integron (8). Comparison of frequency of MDR isolates and presence of class 1 integron in two groups of isolates showed that 92.5% of imipenem non-susceptible isolates were MDR and 91% were positive for class 1 integron. This could be explained with the fact that several studies have reported the presence of different resistance genes, including aminoglycosides, in class 1 integron (8).

In conclusion, the high prevalence of antimicrobial resistance observed among *P. aeruginosa* isolates underlines the strict consideration in antibiotics use at clinical settings. Therefore, it is important to perform antibiotic surveillance programs for appropriate empirical therapy and infection control practices.
